# An Extensive Comparative Analysis of Successful and Unsuccessful Football Teams in LaLiga

**DOI:** 10.3389/fpsyg.2019.02566

**Published:** 2019-11-08

**Authors:** Diego Brito de Souza, Roberto López-Del Campo, Hugo Blanco-Pita, Ricardo Resta, Juan Del Coso

**Affiliations:** ^1^Exercise Physiology Laboratory, Camilo José Cela University, Madrid, Spain; ^2^Department of Competitions and Mediacoach, LaLiga, Madrid, Spain; ^3^Centre for Sport Studies, Rey Juan Carlos University, Fuenlabrada, Spain

**Keywords:** sports performance, match analysis, professional football, soccer, player

## Abstract

The characterization of the in-game actions with the strongest influence on victory in football might be useful for designing playing styles that enhance teams’ performance. The aim of this study was to analyze in-game match statistics on the top-3 and bottom-3 teams ranked in LaLiga. Accumulated offensive and defensive match statistics when playing at home and away were obtained from LaLiga for 8 consecutive seasons. Data extraction was performed by computerized video-analysis. The top-3 and bottom-3 teams were compared using independent *t*-test analysis and the magnitude of the difference was cataloged with effect sizes. Overall, the offensive variable with the greatest magnitude of difference in the top-3 *vs.* bottom-3 comparison was shooting accuracy (ES ± 90% confidence interval = 4.15 ± 0.52) followed by the number of offsides (2.25 ± 0.60) and corners (2.14 ± 0.61). However, when playing away, the offensive variable with the greatest magnitude of difference in the top-3 *vs* bottom-3 comparison was the number of shots (3.30 ± 0.44). The defensive variables that best differentiated top 3 - bottom 3 teams were the number of corners (2.16 ± 0.43) and shots conceded (2.04 ± 0.39). In conclusion, the match statistics that best discriminated successful from unsuccessful football teams were shooting accuracy while attacking and the number of shots conceded while defending.

## Introduction

In this highly technological world, video analysis systems applied to sports performance have become an indispensable tool for coaches and technical assistants to collect information about individual’s and team’s activities during training and competitions (Den Hollander et al., 2018). Specifically, video analysis in football has helped to investigate several aspects of football performance such as technical, tactical, and physiological factors during competition (Sarmento et al., 2014). The speed and accuracy of current computerized video analysis has made possible to scrutinize the in-game actions of elite football teams in real time. Furthermore, the data obtained with this tool has improved the planning and structuring of match and the training programes of high performance teams (Sarmento et al., 2018a). Furthermore, video analysis also constitutes an essential tool for research in team sports and several studies have been published in recent years to aid in understanding football performance, allowing an enhanced application of science to modern football (Lepschy et al., 2018; Sarmento et al., 2018c).

From a simplistic perspective, football performance is an easy concept because victory in this sport is merely based on the comparison of the number of goals scored by each of the two opposing teams at the end of the game. However, a solid body of research has been carried out to determine what aspects of football match-play increase the likelihood of scoring more goals, while avoiding rivals scoring. In order to solve this question, investigations on football performance analysis have compared all types of match statistics between successful and unsuccessful football teams (Oberstone, 2009; Rampinini et al., 2009; Castellano et al., 2012; Liu et al., 2015; Casal et al., 2019). Although extrinsic factors, such as home advantage (Seçkin and Pollard, 2010; Almeida et al., 2014), have been associated with football performance, comparative (Rampinini et al., 2009; Lago-Peñas et al., 2010), and predictive analysis of the statistics during play (Lago-Peñas et al., 2016) have reflected that several in-game actions are the strongest contributors to overall football success.

In a recent review by Lepschy et al. (2018), shooting accuracy was identified as the in-game action that best explained football performance, followed by other offensive variables such as the number of shots on goal, the percentage of ball possession and the rate of passing accuracy. During an elite football match, the success of an offensive sequence is higher when it starts with a counterattack/fast attack in comparison with a positional attack (Sarmento et al., 2018c). In addition, the number of passes performed in an offensive sequence decreases the probability of its success (Sarmento et al., 2018c) coinciding with the low contribution of the number of passes to the points obtained at the end of the season (Souza et al., 2019). A high proportion of the studies included in the review by Lepschy et al. (2018) did not consider the match location although this variable might greatly influence the players’ technical actions that lead to victory (Liu et al., 2015). Furthermore, in a critical review by Mackenzie and Cushion (2013), these authors have suggested that the analysis of in-game actions to predict football performance should be contextualized by match location and thus, a clear identification of the in-game actions that might increase the likelihood of victory is necessary when playing at home *vs* when playing away.

As might be expected, previous investigations have found a clear discrepancy between successful and unsuccessful football teams in highly competitive championships around the world (Rampinini et al., 2009; Lago-Peñas et al., 2011; Liu et al., 2016a). However, these studies have analyzed in-game football actions obtained in only one season, while the year-to-year variability due to players’ injuries or an outstanding player, and the game style imposed by the teams’ coaches might have affected the outcomes of these analyses (Kattuman et al., 2019). In addition, the comparison made in these investigations rarely assesses the importance of each match statistic for overall football performance. The aim of this investigation was to perform a comprehensive comparative analysis of successful and unsuccessful football teams in the Spanish professional football championship (LaLiga) by including accumulated match statistics obtained during 8 competitive seasons. This analysis has considered match location, offensive and defensive event variables and contains a magnitude analysis to improve the work of football coaches and performance analysts.

## Materials and Methods

The current investigation represents a descriptive, comparative analysis of the end of season accumulated match statistics of professional football teams competing in LaLiga (20 teams per season). This research was inspired by a previous investigation carried out by Liu et al. (2016a) using match statistics from LaLiga 2012-2013 but the power of the analysis has been increased with the inclusion of 8 consecutive seasons (from 2010-2011 to 2017-2018). Data were obtained from LaLiga, which owns a software for video match analysis (Mediacoach^®^) based on the OPTA^®^ (Spain) track analysis tool. During the matches, every in-game action is categorized by a mix software tool that included an automatized categorization of some actions by a computerized system (e.g., passes) and categorization by a trained analyst who uses a rigid set of definitions (e.g., yellow and red cards; Liu et al., 2013). The reliability of this current tracking system was tested with an intra-class correlation coefficient that ranges from 0.88 to 1.00 (Liu et al., 2013). To comply with LaLiga ethical guidelines, the information included in this investigation does not allow the recognition of football players’ identities. LaLiga authorized the use of these data for the purpose of this investigation and the experimental protocols were approved by the University Institutional Review Board.

Although 20 professional teams compete in LaLiga every season, we have selected the top 3 and bottom 3 ranked teams at the end of each season in order to perform a comparative analysis of successful and unsuccessful teams. Thus, this study contains information about 24 successful football teams (the top 3 ranked teams for the seasons: 2010-2011, 2011-2012, 2012-2013, 2013-2014, 2014-2015, 2015-2016, 2016-2017 and 2017-2018) and 24 unsuccessful football teams (the bottom 3 ranked teams for the seasons: 2010-2011, 2011-2012, 2012-2013, 2013-2014, 2014-2015, 2015-2016, 2016-2017 and 2017-2018). The match statistics included in this study were based on previous studies with similar aims (Oberstone, 2009; Rampinini et al., 2009; Lago-Peñas et al., 2010; Liu et al., 2015) and they were categorized as offensive and defensive variables to improve the applicability of these results to professional football. A more detailed definition of the match statistics included in this investigation has been published elsewhere (Souza et al., 2019) while the operational definitions for each variable were as follow:

Shot: an attempt to score a goal; shooting accuracy: number of goals divided by the number of shots; pass: an attempt to exchange the ball between two players of the team; passing accuracy: number of successful passes divided by the total number of passes; cross: an action made by a player with the objective of introducing the ball within the opposition team; penalty kick: a single shot on the goal while it is defended only by the opposing team’s goalkeeper; turnover: a loss of ball possession as the result of an imprecision; foul received: an infringement committed by the opposing the team and sanctioned by the referee; corner: an action when the ball crosses the end line of opponent’s side and the last person in contact with the ball was an opponent; free kick goal: a goal scored for the attacking team as the result of a direct or indirect free kick; offside: an infringement committed by the attacking team as a result of a player being offside.

Shot conceded: an attempt to score a goal made by the opposing team; effectiveness against conceded shooting: number of goals received divided by the number of shots conceded; foul committed: an infringement committed by the defending team and sanctioned by the referee; penalty kick conceded: a single shot on the goal while it is defended only by the defending team’s goalkeeper; corner against; an action ball crosses the end line of defending team’s side and the last person in contact with the ball was an opponent; yellow card: a sanction by the referee to one of the players of the defending team; red card: a sanction by the referee to one of the players of the defending team that ends in player expulsion; free kick goals received: a goal received as the result of a direct or indirect free kick; recovery: an action where the team obtains or regains the ball possession due to a defensive action.

To complete the information of this analysis, the end of season accumulated statistics were obtained and subsequently divided into matches played at home and away to allow a sub-analysis for match location.

### Statistical Analysis

The data were electronically extracted from the Mediacoach reports and entered into a database designed for the purposes of this research. The data were extracted by one author (RLDC) using a spreadsheet (Excel 2016, Microsoft Office, WA, United States) and then they were checked for accuracy by another author (DBS). Then, data on the top 3 and bottom 3 football teams in each season were clustered and mean and standard deviation (SD) were obtained. The comparison between the top 3 and bottom 3 teams was performed with independent Student’s *t*-test and the differences were considered as statistically relevant at *P* < 0.05. To complete the null-hypothesis statistical approach, the effect size (ES) was also calculated in all pairwise comparisons to assess the magnitude of the differences between the top 3 and bottom 3 ranked teams. Specifically, the ES ± 90% confidence intervals (CI) were calculated on log transformed data to reduce bias due to non-uniformity of error. A qualitative descriptor was included to represent the likelihood of differences among teams (< 1% no chances of change; 1 to 5%, very unlikely; 5 to 25%, unlikely; 25 to 75%, possible; 75 to 95%, likely; 95 to 99%, very likely; >99%, most likely). ES were interpreted according to the following ranges:<0.2, trivial; 0.2–0.6, small; 0.6–1.2, moderate; 1.2–2.0, large; 2.0–4.0, very large; and >4.0, extremely large (Hopkins et al., 2009). This same analysis was performed for the overall accumulated statistics and for the games played at home and away.

## Results

**FIGURE 1:**
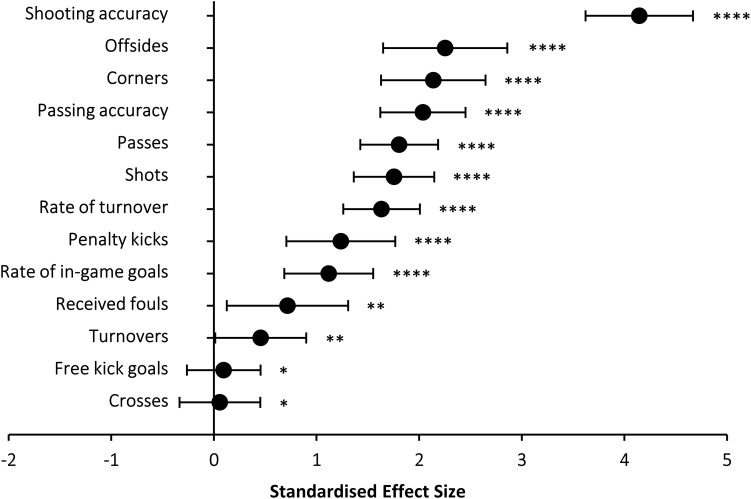
Standardized effect size in offensive variables for the comparison between the top 3 – bottom 3 teams in LaLiga from 2010 to 2018. ^∗^possible; ^∗∗^likely; ^****^most likely.

Figure 1 and Table 1 contain data on the compariso’n between top 3 and bottom 3 teams for the offensive in-game actions. In all offensive actions, a statistically significant between-group difference was identified at the level of *P* < 0.01. However, the ES have been included to improve the categorization of the magnitude of the successful *vs.* unsuccessful teams’ differences: overall, shooting accuracy was the offensive variable with the highest effect size for the top 3 - bottom 3 comparison (Figure 1) while the importance of shooting accuracy to differentiate top 3 *vs.* bottom 3 teams was maintained when playing at home and when playing away (Table 1). When attacking, the number of offsides and corners, passing accuracy, and the total number of passes presented large effect sizes for the top 3- bottom 3 comparison, with a most-likely difference among teams (Figure 1). At home, shooting accuracy, the number of offsides, passing accuracy and the number of corners also presented large effect sizes for the top 3 - bottom 3 comparison, while the number of free kick goals and the number of crosses were the variables with the lowest effect sizes. When playing away, the total number of shots was the variable with the greatest effect size, followed by shooting accuracy and the number of passes and passing accuracy (Table 1).

**TABLE 1:** Attacking variables for the top 3 and bottom 3 teams ranked in LaLiga from 2010 to 2018.

		**Home**			**Away**			**Total**	
			
**Offensive variables**	**Top 3**	**Bottom 3**	**ES (90%CI)**	**Top 3**	**Bottom 3**	**ES (90%CI)**	**Top 3**	**Bottom 3**	**ES (90%CI)**
Shooting accuracy (%)	16 ± 2	8 ± 1	4.15 (0.52)	15 ± 3	8 ± 2	2.76 (0.49)	16 ± 2	8 ± 1	4.15 (0.52)
Offsides (number)	64 ± 8	47 ± 12	2.46 (0.75)	59 ± 14	42 ± 10	1.48 (0.49)	123 ± 17	89 ± 20	2.25 (0.60)
Corners (number)	136 ± 15	106 ± 15	2.23 (0.53)	103 ± 18	78 ± 17	1.40 (0.48)	239 ± 27	184 ± 26	2.14 (0.61)
Passing accuracy (%)	83 ± 5	73 ± 3	2.33 (0.44)	81 ± 5	72 ± 3	1.70 (0.41)	82 ± 5	72 ± 3	2.04 (0.42)
Passes (number)	11712 ± 1998	8379 ± 795	1.88 (0.38)	10986 ± 2065	7814 ± 837	1.72(0.38)	22698 ± 4036	16193 ± 1560	1.81 (0.38)
Shots (number)	328 ± 54	243 ± 25	1.65 (0.39)	328 ± 54	183 ± 28	3.30 (0.44)	594 ± 102	427 ± 47	1.76 (0.39)
Rate of turnover (%)	21 ± 4	31 ± 3	1.86 (0.38)	23 ± 5	31 ± 3	1.36 (0.37)	22 ± 5	31 ± 3	1.63 (0.37)
Penalty kicks (number)	5 ± 3	3 ± 2	0.68 (0.47)	3 ± 2	2 ± 1	1.08 (0.50)	8 ± 4	4 ± 3	1.24 (0.53)
Rate of in-game goals (%)	83 ± 7	74 ± 6	1.12 (0.43)	83 ± 7	74 ± 12	1.13 (0.63)	83 ± 7	74 ± 6	1.12 (0.43)
Received fouls (number)	260 ± 24	259 ± 30	0.07 (0.53)	287 ± 31	260 ± 36	0.91 (0.52)	547 ± 42	518 ± 61	0.72 (0.59)
Turnovers (number)	2402 ± 172	2583 ± 141	0.99 (0.42)	2420 ± 152	2382 ± 177	0.25 (0.51)	4822 ± 301	4965 ± 279	0.46 (0.44)
Free kick goals (number)	17 ± 7	26 ± 6	1.12 (0.42)	26 ± 12	26 ± 6	0.06 (0.63)	17 ± 7	16 ± 2	0.10 (0.36)
Crosses (number)	426 ± 107	445 ± 63	0.24 (0.37)	342 ± 72	322 ± 64	0.22 (0.43)	768 ± 168	767 ± 118	0.06 (0.39)

*The effect size (ES) has been calculated for the difference between the top 3 – bottom 3 teams when playing at home, away and overall. Data are mean ± SD for the data at the end of the season.*

**FIGURE 2:**
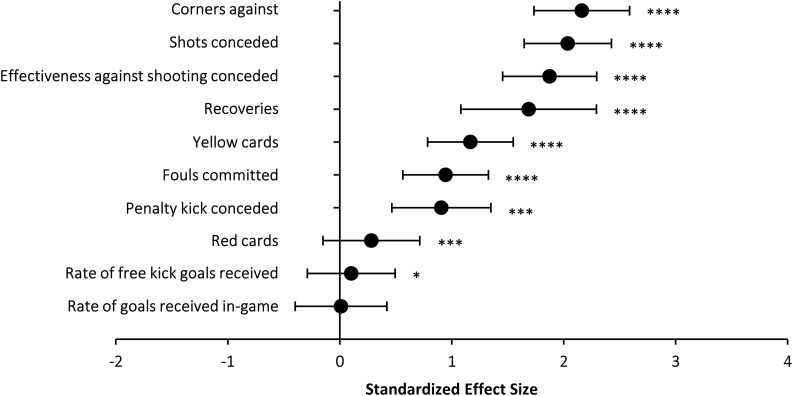
Standardized effect size in defensive variables for the comparison between the top 3 – bottom 3 teams in LaLiga from 2010 to 2018. ^∗^possible; ^∗∗∗^very likely; ^****^most likely.

Figure 2 and Table 2 depict data on the comparison of the top 3 *vs.* bottom 3 teams for all defensive game actions. Again, all defensive match statistics presented between-group differences at the level of *P* < 0.01. However, the match statistics with the highest effect size for the comparison of successful and unsuccessful teams were the number of corners, the number of shots conceded and the effectiveness against shooting conceded (Figure 2). On the contrary, the number of red cards, and the distribution of in-game/free kick goals received were the variables with the lowest effect sizes for the comparison between top 3 *vs.* bottom 3 teams. At home, the number of shots conceded was the variable with the greatest effect size (Table 2). When playing away, the number of corners conceded was the variable with the highest effect size (Table 2). Other variables such as recoveries, yellow cards and fouls committed also presented large and most-likely differences between successful and unsuccessful teams (Figure 2) and the effect sizes of these variables slightly changed for the comparisons made at home and away.

**TABLE 2:** Defensive variables for the top 3 and bottom 3 teams ranked in LaLiga from 2010 to 2018.

		**Home**			**Away**			**Total**	
			
**Defense variables**	**Top 3**	**Bottom 3**	**ES (90%CI)**	**Top 3**	**Bottom 3**	**ES (90%CI)**	**Top 3**	**Bottom 3**	**ES (90%CI)**
Corners against (number)	69 ± 14	95 ± 17	1.55 (0.43)	87 ± 15	124 ± 16	2.11 (0.42)	156 ± 24	219 ± 28	2.16 (0.43)
Shots conceded (number)	167 ± 30	231 ± 23	1.79 (0.38)	216 ± 32	292 ± 29	2.03(0.40)	383 ± 57	523 ± 48	2.04 (0.39)
Effectiveness against shooting conceded (%)	9 ± 2	12 ± 3	1.21 (0.42)	8 ± 2	14 ± 3	1.92(0.44)	9 ± 2	13 ± 2	1.87 (0.42)
Recoveries (number)	1015 ± 60	929 ± 91	1.49 (0.64)	985 ± 73	871 ± 87	1.61 (0.55)	2000 ± 122	1800 ± 169	1.69(0.61)
Yellow cards (number)	37 ± 10	55 ± 8	1.36 (0.38)	51 ± 12	57 ± 8	0.61 (0.37)	88 ± 20	113 ± 14	1.17 (0.38)
Fouls committed (number)	236 ± 38	274 ± 29	0.98(0.40)	236 ± 44	273 ± 24	0.84 (0.37)	472 ± 78	547 ± 48	0.95 (0.38)
Penalty kick conceded (number)	1 ± 1	2 ± 1	0.99 (0.54)	2 ± 2	4 ± 2	0.71 (0.52)	3 ± 2	6 ± 2	0.91 (0.44)
Red cards (number)	1 ± 1	2 ± 1	0.08 (0.56)	1 ± 2	1 ± 1	0.02 (0.50)	2 ± 2	3 ± 1	0.28 (0.43)
Free kick goals received (%)	21 ± 10	20 ± 8	0.14 (0.44)	20 ± 8	21 ± 7	0.07 (0.49)	20 ± 7	20 ± 5	0.10 (0.39)
Goals received in game (%)	79 ± 10	80 ± 8	0.12 (0.42)	80 ± 8	79 ± 7	0.17(0.45)	80 ± 7	80 ± 5	0.01 (0.41)

*The effect size (ES) has been calculated for the difference between the top 3 – bottom 3 teams when playing at home, away and overall. Data are mean ± SD for the data at the end of the season.*

## Discussion

The aim of this investigation was to perform a comparative analysis of successful and unsuccessful football teams in the LaLiga championship. Although the comparison of successful/winning *vs.* unsuccessful/losing football teams has been previously explored (Lago, 2009; Lago-Peñas et al., 2010; Liu et al., 2016a), the current analysis is innovative because it includes accumulated match statistics obtained during 8 competitive seasons, which constitutes the longest analysis on this topic. In addition, the comparison between successful and unsuccessful teams has taken into account match location and used two different statistical approaches to categorize the contribution of each in-game action to overall football performance. The main outcomes of this investigation reflect that the in-game actions that differentiate the top 3 and bottom 3 football teams in LaLiga were very similar when playing at home and away (Tables 1, 2) which reflects that success in football might be driven by a similar game-play style despite match location. Offensively, the match statistic with the greatest difference, in terms of effect size, between the top 3 and bottom 3 football teams was shooting accuracy. Defensively, the greatest difference between best *vs* worse teams was the number of corners received. Taken together, these outcomes indicate that modern football has evolved from “long-ball” to more direct playing styles where long passing frequency might not be better for scoring (Hughes and Franks, 2005). All this information might be useful to define success in elite Spanish football and help coaches and football analysts to understand the strategy followed by top-ranked teams that compete in one of the most important football championships (Vales-Vázquez et al., 2017).

In a study of 380 matches in LaLiga –season 2008-2009–, it was found that top-ranked teams scored more goals, shot more frequently, particularly on goal, and needed less opportunities than worse-ranked teams (Lago-Ballesteros and Lago-Peñas, 2010). In addition, in a study with 3,040 matches in LaLiga, shooting accuracy was the variable that explained more variance in the end-season points earned during the championship (Souza et al., 2019). The current analysis coincides in part with these investigations because the variable showing the greatest difference between the top 3 and bottom 3 football teams was shooting accuracy (Figure 1). Interestingly, a similar finding has been obtained in the Bundesliga (Broich et al., 2014), the Superleague in China (Mao et al., 2016), and when analyzing the final rounds of the European Champions League (Szwarc, 2007), and the 2010 World Cup (Delgado-Bordonau et al., 2013). Although the results of this investigation cannot be generalized to all football situations and competitions, the clear importance of shooting accuracy might impact tactical-strategic aspects of elite teams’ training.

Shooting accuracy was followed by the number of offsides and corners which, despite not being direct shooting actions, are reflective of a game style focused on direct play to score. As found by others (Lago-Peñas, Lago-Ballesteros, Dellal, et al; Lago-Peñas, Lago-Ballesteros, and Rey), one of the characteristics of successful teams is that they create more attack opportunities especially in the field area close to the opponents’ goal. Although the current investigation constitutes an analysis of 8 complete seasons to produce a study with a high statistical power, it is worth mentioning that the season-to-season analysis reflects that the match statistics with the greatest difference between the top 3 and bottom 3 football teams were fairly maintained during the whole period analyzed. Other match statistics such as passes and passing accuracy also presented high effect sizes for the top 3 - bottom 3 comparison while other offensive factors such as free kick goals and crosses were even higher in the bottom 3 teams. Although the significance of these data is debatable, in the opinion of the authors of this investigation, passing should be to gain offensive zones and with the clear intention of attacking, and providing opportunities for scoring. Recent studies have also found that successful teams use ball possession to attack while unsuccessful teams tend to use possession to avoid losing the ball (Casal et al., 2019).

One of the most novel findings of this investigation is that the offensive match statistics that best differentiate the top 3 and bottom 3 football teams were very similar when the teams played at home and away (Table 1). Nevertheless, subtle nuances are found; overall, shooting accuracy was key to success and this criterion was maintained when playing at home but the number of shots was even more important when playing away. In addition, the magnitudes of the effect sizes for the top 3 – bottom 3 comparison in all shooting and passing variables were higher at home than away, which suggests that the difference between successful and unsuccessful teams in offensive variables might increase with match location (Lago and Martín, 2007). Although match location, quality of opposition, and match status should be useful to adapt game tactics (Liu et al., 2016b), the current data on successful teams in LaLiga suggest that the main objective of offensive strategy –obtaining clear situations for shooting- should be maintained when playing at home and away.

Although less attention has been paid to defensive variables (Mackenzie and Cushion, 2013), the current analysis indicates that successful and unsuccessful team are also very different in terms of defense match statistics (Figure 2). Overall, the number of corners received was the variable with the highest effect size in the top 3 – bottom 3 comparison, even above the number of shots conceded. Although this might be a particularity of this analysis, the high rate of corners conceded is a common finding in worse-ranked teams (Castellano et al., 2012). Broadly, only 2% of the corners end in goal but the influence of a goal obtained from a corner might determine victory in < 75% of the games (Casal et al., 2015). Effectiveness against rivals’ shooting also presented a large between-group difference but in this case, this match statistics was better in unsuccessful teams both at home and away. This means that the defensive efficacy of worse-ranked teams is not inferior to top-ranked teams but the former offer more opportunities for attack to the opposing team (Delgado-Bordonau et al., 2013; Evangelos et al., 2013).

Despite the high statistical power obtained by the accumulated match statistics of 8 football seasons, the current research does have some limitations. Previous investigations have identified that the quality of the opposing team, players’ physical conditioning and several contextual variables are strongly related to a team’s success in football (Taylor et al., 2008; Lago, 2009; Liu et al., 2016a; Sarmento et al., 2018b). Although the current analysis does not include these variables, it is likely that their influence on the outcomes of the analysis are minimal due to the use of eight complete seasons of a professional league, that includes the same number of matches and the same rivals for all the teams under investigation. A second limitation is that the pitch area where the in-game action occurred was not recorded for this investigation and further research should be done to relate the outcomes of this study with pitch zones (Lepschy et al., 2018; Sarmento et al., 2018a). Despite these limitations, the findings of this analysis might contribute to understanding success in football.

This investigation has been carried out with the intention of identifying key game indicators that differentiate successful and unsuccessful teams to determine a more effective model of play. In summary, the study of the top 3 and bottom 3 ranked teams in LaLiga for 8 seasons might be indicative of a sport where shooting accuracy prevails over other offensive statistics. While all the attacking game actions investigated here were statistically higher in the top 3 teams *vs* bottom 3 teams, those performed close to the penalty area presented higher effect sizes (see Figure 1). Regarding defensive game actions, the number of corners and the number of shots conceded were the variables with the highest differences in terms of size between successful and unsuccessful teams. While a greater efficacy against rival’s shooting was present in worse-ranked teams, it is probably due to the higher number of shots received. In this sense, it is probably necessary that less successful teams enhance game tactics or change their playing style to avoid/reduce rival’s shooting during the match. Finally, the identification of the game statistics related to success was stable when comparing matches played at home and away, suggesting that a similar game style should be maintained despite match location in order to maximize football performance.

To improve football performance of elite teams, several practical applications can be gathered from the data obtained in this investigation. Specifically, the use of training routines that improve shooting accuracy might be essential to increase the efficacy of offensive sequences. The use of fast attacking routines (with and without opponents) with the main objective of shooting on goal with a low number of passes might be recommended to improve offensive behaviors of the team. The use of exercises that improve tactics during corners (when attacking and defending) might be also critical for football performance as this game action is a clear indicator that differentiated successful and unsuccessful teams in LaLiga. Finally, tailoring training exercises to improve the recovery of the ball before the rival team reaches offensive positions might be also beneficial to avoid the number of shots conceded while defending.

## Data Availability Statement

The datasets generated for this study are available on request to the corresponding author.

## Ethics Statement

The studies involving human participants were reviewed and approved by Camilo José Cela Institutional Review Board. Written informed consent for participation was not required for this study in accordance with the national legislation and the institutional requirements.

## Author Contributions

RL-D, HB-P, and RR provided the data. DB and JD carried out the analysis of the data and drafted the manuscript. RL-D, HB-P, and RR critically reviewed the manuscript. All authors have participated in the conception of the experiment and approved the final version of the manuscript.

## Conflict of Interest

RL-D, HB-P, and RR were LaLiga employees during the preparation of this work. The remaining authors declare that the research was conducted in the absence of any commercial or financial relationships that could be construed as a potential conflict of interest.
